# Sanguinarine protects against indomethacin-induced small intestine injury in rats by regulating the Nrf2/NF-κB pathways

**DOI:** 10.3389/fphar.2022.960140

**Published:** 2022-10-11

**Authors:** Xiu-lian Lin, Ya-ning Shi, Yu-ling Cao, Xi Tan, Ya-ling Zeng, Shi-teng Luo, Ya-mei Li, Li Qin, Bo-hou Xia, Rong-geng Fu, Li-mei Lin, Kai Li, Deliang Cao, Jian-guo Zeng, Duan-fang Liao

**Affiliations:** ^1^ Key Laboratory for Quality Evaluation of Bulk Herbs of Hunan Province, Hunan University of Chinese Medicine, Changsha, Hunan, China; ^2^ Division of Stem Cell Regulation and Application, Hunan University of Chinese Medicine, Changsha, Hunan, China; ^3^ Hunan Key Laboratory of Traditional Chinese Veterinary Medicine, Hunan Agricultural University, Changsha, Hunan, China

**Keywords:** sanguinarine, small intestine injury, indomethacin, Nrf2/NF-κB pathways, rats, IEC-6 cells

## Abstract

In recent years, small intestine as a key target in the treatment of Inflammatory bowel disease caused by NSAIDs has become a hot topic. Sanguinarine (SA) is one of the main alkaloids in the *Macleaya cordata* extracts with strong pharmacological activity of anti-tumor, anti-inflammation and anti-oxidant. SA is reported to inhibit acetic acid-induced colitis, but it is unknown whether SA can relieve NSAIDs-induced small intestinal inflammation. Herein, we report that SA effectively reversed the inflammatory lesions induced by indomethacin (Indo) in rat small intestine and IEC-6 cells in culture. Our results showed that SA significantly relieved the symptoms and reversed the inflammatory lesions of Indo as shown in alleviation of inflammation and improvement of colon macroscopic damage index (CMDI) and tissue damage index (TDI) scores. SA decreased the levels of TNF-α, IL-6, IL-1β, MDA and LDH in small intestinal tissues and IEC-6 cells, but increased SOD activity and ZO-1 expression. Mechanistically, SA dose-dependently promoted the expression of Nrf2 and HO-1 by decreasing Keap-1 level, but inhibited p65 phosphorylation and nuclear translocation in Indo-treated rat small intestine and IEC-6 cells. Furthermore, in SA treated cells, the colocalization between p-p65 and CBP in the nucleus was decreased, while the colocalization between Nrf2 and CBP was increased, leading to the movement of gene expression in the nucleus to the direction of anti-inflammation and anti-oxidation. Nrf2 silencing blocked the effects of SA. Together our results suggest that SA can significantly prevent intestinal inflammatory lesions induced by Indo in rats and IEC-6 cells through regulation of the Nrf2 pathway and NF-κBp65 pathway.

## Introduction

Non-steroidal anti-inflammatory drugs (NSAIDs), such as aspirin, diclofenac, indomethacin, and celecoxib, are widely prescribed for treatment of pain and inflammation in a variety of chronic conditions, such as rheumatoid arthritis and osteoarthritis. One of the major adverse effects of NSAIDs is severe gastrointestinal complications, such as hemorrhage, ulceration and perforation (Watanabe et al., 2020). The introduction of new modalities, such as capsule endoscopy (Iddan et al., 2000) and balloon-assisted endoscopy (Matsumoto et al., 2008) revealed that NSAIDs result not only in colitis, but also in small bowel lesions, including various types of mucosal damage, such as petechiae, red spots, erosions, and ulcers (Graham et al., 2005; Maiden et al., 2005), which often limits the use of NSAIDs (Watanabe et al., 2020).

Inflammatory bowel disease (IBD) includes ulcerative colitis (UC) and Crohn’s disease (CD). The former mainly affects the colorectum, while the latter affects the large intestine and small intestine. Therefore, previous studies on IBD focused more on the colon (Watanabe et al., 2020). In recent years, International journals such as Nature, Science and Cell have reported that the small intestine is involved in the enterohepatic circulation (Chen et al., 2021) and gut-brain-axis regulation (Bauer et al., 2016). In particular, the intestinal microbes play a key role in regulation of host immune function (Kim et al., 2016; Schluter et al., 2020; Chen et al., 2021; Wastyk et al., 2021). In particular, Chen et al. (2021) found that the small intestine, especially small intestine lamina propria (siLP), is the place of the CAR/MDR1 pathway activating, transcriptional reprogramming and sub-specialization of Teff cells, and hereby Teff cells prevents against bile acid toxicity and inflammation in the mouse small intestine. On the other hand, the intestinal microbes in small bowel play a key role in regulation of host immune function (Kim et al., 2016; Schluter et al., 2020; Wastyk et al., 2021). The small intestine is also closely associated with inflammation caused by NSAIDs (Leung et al., 2007). These studies strongly suggest the role of small intestine as a key target in the prevention and treatment of human intestinal diseases.

Small intestinal mucosal barriers (SIMB) include physical barrier, chemical barrier, immune barrier and biological barrier. The physical barrier, consisting mainly of a single layer of small intestinal epithelial cells (IECs) and tight junctions (TJs), plays an important role in NSAIDs-induced intestinal lesions. SIMB damage was recently recognized as common complications of NSAIDs. Goldstein, et al. reported that small bowel mucosal breaks occurred in 55% of healthy volunteers given naproxen for 2 weeks (Graham et al., 2005). Matsumoto, et al. showed that the intestinal ulceration occurred in around 51% patients taking NSAIDs (Matsumoto et al., 2008). Similarly, 2-week ingestion of slow-release diclofenac resulted in intestinal ulceration in 68%–75% of healthy volunteers (Maiden et al., 2007).

It is generally recognized that the pathologic changes of NSAIDs-induced enteritis are caused mainly by inflammatory and oxidative stress. Existing literature suggests that SIMB injuries, in particular endothelial cell damage, are the main pathologic changes, in which inflammatory stress plays a key role (Maiden et al., 2007; Watanabe et al., 2020). Therefore, control of the inflammation of small intestinal mucosa is the main strategy to mitigate intestinal damage in NSAIDs users (Lanza et al., 2009). It has been reported that many agents, such as misoprostol (Taha et al., 2018), rebamipide (Kurokawa et al., 2014), rifaximin (Fornai et al., 2016), and probiotics (Endo et al., 2011), can alleviate intestinal damage induced by NSAIDs. Nevertheless, clinical trials have not demonstrated that any of these drugs are clearly effective for treatment of NSAID-induced small bowel lesions other than drug withdrawal (Watanabe et al., 2020).

Botanical drugs and Chinese medicine have unique advantages and prospects in relieving the NSAIDs-induced damage of small intestinal mucosa. It has been reported that berberine (He et al., 2018), curcumin (Wang et al., 2021), quercetin (Fan et al., 2021), all showed obvious protective effects on NSAIDs-induced intestinal mucosal damage.

Sanguinarine (SA) is a benzophenanthridine alkaloid derived from the roots of *Sanguinaria canadensis* or other poppy-fumaria species. SA also exits in the seeds of *Argemone mexicana* L. and the capsules of *Macleaya cordata* (Lin et al., 2020). SA is present in the cationic iminium and neutral alkanolamine forms, and a positive moiety exits in the aromatic ring of the molecule. Many studies have shown that SA has impressive biological activity and drug developmental potentials, especially for the treatment of chronic human diseases, such as cancer and asthma (Basu and Kumar, 2016).

Several studies demonstrated that *Macleaya cordata* extracts improved gut health of early-weaned piglets (Chen et al., 2018), alleviated oxidative damage induced by weaning in the lower gut of young goats (Chen K. et al., 2020), and improved intestinal barrier function in growing piglets (Liu et al., 2016). It has been reported that SA has protective effects in acetic acid-induced ulcerative colitis in mice (Niu et al., 2013). However, whether SA can relieve small intestine inflammatory injuries induced by NSAIDs has not been reported. This study unraveled for the first time that SA could prevent intestinal epithelial cells from NSAIDS-induced inflammatory lesions by regulating the Nrf2/NF-κB pathways.

## Materials and methods

### Materials

Sanguinarine and berberine were purchased from Beijing Solarbio Science & Technology Co., Ltd. and Shanghai Yuanye Bio-Technology Co., Ltd., respectively. Indomethacin was purchased from MedChemExpress Bio-Technology Co., Ltd., United States. Antibodies against Keap-1 (ab119403), Nrf2(ab89443 and ab62352), HO-1 (ab189491 and ab68477), Claudin-1 (ab180158) and ZO-1 (ab276131) proteins were purchased from ABCOM; CBP (#7389), NF-κB p65 (#8242) and phospho-NF-κB p65 (#3033) were purchased from Cell Signaling Technology. Phospho-NF-κB p65 (sc-136548), Nrf-2 (sc-365949) were purchased from Santa Cruz Biotechnology, Inc. β-actin (20536-1-AP), GAPDH (60004-1-Ig), horseradish peroxidase (HRP)-conjugated goat anti-rabbit antibody (SA00001-2), HRP-conjugated goat anti-mouse antibody (SA00001-1), and Alexa Fluor 488-conjugated goat anti-mouse antibody (SA00013-1) were products of Proteintech Co., Ltd. Cy3 conjugated Goat anti-Rabbit IgG (H+L) (GB21303) and Cy3 conjugated Donkey anti-Rabbit IgG (H+L) (GB21403) were purchased from Servicebio Co., Ltd.

Kits of tumor necrosis factor-α (TNF-α), interleukin-6 (IL-6), and interleukin-1β (IL-1β) were purchased from Wuhan Huamei Biological Engineering Co., LTD. The CCK-8 kit was purchased from BioSharp. LDH, MDA and SOD kits were purchased from Nanjing Jiancheng Institute of Biological Engineering. DMEM (Zq-100), 10% newestern blotorn bovine serum (AU0600), 1%P/S (CSP006), and insulin (10 μg ml^−1^, Csp001-10) were all purchased from Shanghai Zhongqiao Xinzhou Biotechnology Co., Ltd.

Three types of Nrf2 siRNA were provided by Sangon Biotech (Shanghai) Co., Ltd. Their sequences are as follows:

1. R Nrf2α-539: sense (5′-3′) GCC​AGG​CCA​UAG​ACA​UCA​ATT, antisense (5′-3′) UUG​AUG​UCU​AUG​GCC​UAA​CTT
2. R Nrf2α-869: sense (5′-3′) CCA​AGC​AUA​UCA​CAA​CCA​UTT, antisense (5′-3′) AUG​GUU​GUG​AUA​UGC​UUG​GTT
3. R Nrf2α-1470: sense (5′-3′) GCU​AAA​UCA​GCC​UGA​AUU​ATT, antisense (5′-3′) UAA​UUC​AGG​CUG​AUU​UAG​CTT.

### Animals

Male SPF grade clean SD rats at 180–200 g were purchased from Hunan Silaike Jingda Laboratory Animal Co., Ltd. Rats were maintained in an air-conditioned (24 ± 2°C) room with light cycle of 12 h light + 12 h dark and humidity at 50% ± 5% with free access to drink and food. The animal studies were performed in accordance with the Animal Ethics Guide of Experimental Animal Society of China and approved by the Hunan University of Chinese Medicine (SYXK (xiang)2019-0009).

### Development of rat small bowel inflammation induced by indomethacin

According to the literature method (Nandi et al., 2010), a total of 60 male SD rats at 180–200 g were randomly divided into 6 groups (*n* = 10 each): saline control (Ctrl), indomethacin (Indo, 7.5 mg kg^−1^), berberine positive control (Ber, 60 mg kg^−1^), Indo + SA at low-dose (SA-L, 0.33 mg kg^−1^), Indo + SA at medium-dose (SA-M, 1.0 mg kg^−1^) and Indo + SA at high-dose (SA-H, 3.3 mg kg^−1^). After 3 days of adaptive feeding, SA and Ber groups were administered with SA or berberine at indicated doses by gavage for 3 days. Thereafter, except for the Ctrl group delivered with saline, all rats were given indomethacin at 7.5 mg kg^−1^ subcutaneously, once a day for 2 days, SA and Ber were administered simultaneously and continued to be administered by gavage until the ninth day.

### Abdominal cavity macroscopy and colonic mucosa damage index scores

24 h after the last dose of SA or berberine, the rats were deeply anesthetized, the abdominal cavity was opened, and the small intestine effusion, redness, adhesions and necrosis were evaluated and recorded by macroscopy. Jejunum tissues were then excised, washed with ice phosphate buffer (PBS), and longitudinally opened along the mesentery to observe jejunum mucosal ulcers, erosion, hyperplasia and other conditions.

The colonic mucosa damage index (CMDI) scores were evaluated as reported in literature (Gautam et al., 2016; Ke et al., 2019): 0 point: no gross lesions; 1 point: mild hyperemia, edema, smooth surface, but no erosion or ulcers; 2 points: hyperemia, edema, rough and granular mucosa, erosion or intestinal adhesion; 3 points: high hyperemia and edema, mucosal necrosis and ulcer formation, the maximum longitudinal diameter of the ulcer is less than 1 cm, intestinal wall thickening or necrosis and inflammation on the surface; 4 points: high degree of hyperemia and edema, mucosal necrosis and ulceration, the maximum longitudinal diameter of ulceration ≥1 cm, or necrosis of the whole intestinal wall and dilation of the intestinal lumen that may lead to death.

### Histological analysis and tissue damage index scores

Jejunum tissues of rats were taken for routine fixation and paraffin embedding, followed by sectioning and H&E staining and then histologically estimated under light microscope. Tissue damage index (TDI) scores were evaluated according to standards previously reported (Xu et al., 2020). The scoring indexes were set up at 0–3 points according to the degree of lesions, including epithelial injury, ulcer depth, lymphocyte infiltration, edema and infiltration of inflammatory cells (such as neutrophils and eosinophilic cells).

### Cell culture

Rat small intestinal crypt epithelial cells (IEC-6, ZQ0783) were provided by the Cell Bank of the Chinese Academy of Science (Shanghai, China). IEC-6 cells were cultured and maintained in DMEM medium with 10% newestern blotorn bovine serum, 1% penicillin/streptomycin and 10 μg mL^−1^ insulin in a humidified incubator with 5% CO_2_ and 95% O_2_ at 37°C (Fan et al., 2021).

For drug treatment, IEC-6 cells were spread in 6-well plates at a density of 2 × 10^5^ cells/well and pre-treated with SA (0.25, 0.5, and 1.0 μmol L^−1^) or berberine at 30 mmol·L^−1^for 12 h, followed by 300 μmol L^−1^ indomethacin for 24 h. Indomethacin vehicle was used as a control.

### Evaluation of cell viability

IEC-6 cells were incubated in 96-well culture plates at 5.0×10^3^ cells per well for 24 h, followed IEC-6 cells were preincubated with SA (0.25–1.0 μmol L^−1^) in the medium for 12 h, and then indomethacin (300 μmol L^−1^) was added for 24 h of continuing treatment. According to the instructions of the CCK-8 kit, 10% CCK-8 solution was added to the wells (100 μl/well) for 2–2.5 h. The absorbance (OD values) of IEC-6 cells at 450 nm was read using a microplate reader. Cell viability (%) = (OD_treatment_–OD_blank_)/(OD_control_–OD_blank_) × 100%.

### Measurement of lactate dehydrogenase, Malondialdehyde and super oxide dismutase

An appropriate amount of protein from the rat jejunum tissues or IEC-6 cells were processed. The serum was collected from abdominal aorta, placed at 4°Cfor 30 min, and centrifuged at 1000 *g* and the supernatant was taken. The cell culture supernatant was collected directly and centrifuged at 4000 *g* before use. Protein concentrations were quantified by BCA method. The levels of LDH, MDA and SOD were measured and calculated according to the instructions of LDH, MDA and SOD, respectively. LDH/MDA/SOD = (Measured OD value–control OD value)/(standard OD value–blank OD value) × standard concentration/protein concentration of the tested sample.

### Measurement of IL-1β, IL-6 and TNF-α

100 mg of rat jejunum tissues were accurately weighed, added into 500 μl cold PBS and homogenized with a tissue grinding apparatus at 4°C for 3 times with 45 s grinding and 20 s interval each time. After centrifuged at 7000 *g* for 10 min, then the supernatant was collected and protein concentrations were quantified by BCA method. TNF-α, IL-6 and IL-1β were detected in accordance with the ELISA Kit instructions.

### Molecular docking analysis

Molecular docking analysis of Sanguinarine in the kelch pockets of Keap-1 was performed using Gold 3.0. The small ligand-binding C-terminal kelch domain of the human Keap-1 (PDB: 4XMB) was selected as described in previous studies (Georgakopoulos1 et al., 2016; Jain et al., 2015).

### Western blotting

100 mg of tissues from the rat jejunum were taken and cleaned with PBS at 4°C, followed by homogenization in RIPA with protease inhibitors (100:1) at 4°C for 3 times with 45 s grinding and 20 s interval each time. Centrifugation was performed at 16,000 *g* for 15 min, and the supernatant was taken. BCA kit was used for protein quantification. Bromophenol cyanogen loading buffer was added and denatured at 100°C for 10 min. Western blot was performed by SDS-PAGE gel electrophoresis, and the target proteins were isolated by 6%–10% SDS-PAGE electrophoresis, blotted onto the PVDF membrane, and then sealed with 5% milk or 5% BSA (prepared in TBST) for 1 h. Primary antibodies against Keap-1, Nrf2, HO-1, p65, p-p65, CBP or ZO-1 was added and incubated overnight at 4°C. Secondary antibodies corresponding to primary antibody hosts were incubated next day at 37°C for 1 h, and then exposed. The gray values of the target protein bands were calculated using Image J Software.

### Immunofluorescence and colocalization analysis

Jejunum tissues were formalin fixed and paraffin embedded, and 3 mm sections were dewaxed in the following order: xylene I for 15min, xylene II for 15 min, anhydrous ethanol for 15 min, 85% ethanol for 5 min, 75% ethanol for 5 min, distilled water for 5 min.Then the antigen was repaired with EDTA repair solution (pH: 8.0–9.0) and hydrophobic histochemical pen was used to block the hydrosphere. After the slides were sealed in 3%–5% BSA for 30–60 min primary antibody was added and incubated overnight at 4°C, followed by respective secondary antibodies conjugated with fluorescence.

IEC-6 cells were treated with 4% paraformaldehyde for 15 min, then treated with 0.1% Triton/PBS at 4 °C and permeabilized for 10–20 min according to the expression position of the target protein. Then IEC-6 cells were incubated with 3–5% BSA for 1 h followed by primary antibody overnight at 4 °C. On the second day, the jejunum sections and IEC-6 cells were washed with PBS for 3 times, 5min/per time, and then incubated with corresponding fluorescence secondary antibody for 1 h. After washed with PBS 3 times, 5min/per time, DAPI was used to stain the nuclei. Nikon A1R confocal microscope was used for evaluation, Image J and Image-Pro Plus software was used for Image analysis.

### siRNA silencing

The IEC-6 cells were inoculated in antibiotic-free medium, and the number of cells was controlled at 50%–60% confluence 24 h after inoculation. Before siRNA transfection, cells were rinsed with PBS for 3 times, and the medium was replaced with serum-free medium. SiRNA was diluted with Opti-MEM medium (reduced serum) until the final concentration at 50 n mol·L^−1^, and then mixed gently. Lipofectamine2000 was diluted with Opti-MEM and incubated at room temperature for 5 min. The diluted siRNA and lipofectamine2000 were gently mixed and incubated at room temperature for 15 min. The mixture of siRNA and lipofectamine2000 was then added into the medium, and the whole process was completed within 30 min. 4–6 h after transfection with siRNA, the IEC-6 cells were incubated in serum-containing medium. When the cell confluence reached at 90%, the cells were incubated with Ber or SA for 12 h, and then treated with indomethacin for 24 h. Total protein of the cells was extracted, and the expression of related proteins was detected.

### Statistical analysis

Data are displayed as a mean of at least three separate experiments ± standard deviation (SD). For the comparisons between two groups, Student’s t-test was used for independent samples. One-way analysis of variance (ANOVA) test with Dunnett’s method was performed to compare multiple groups. A p-value ≦0.05 was considered statistically significant. GraphPad Prism version 8.0 software (GraphPad Software, Inc., La Jolla, CA, United States) was used for statistical analyses.

## Results

### Protection of Sanguinarine on small bowel inflammatory lesions induced by indomethacin in rats

**FIGURE 1:**
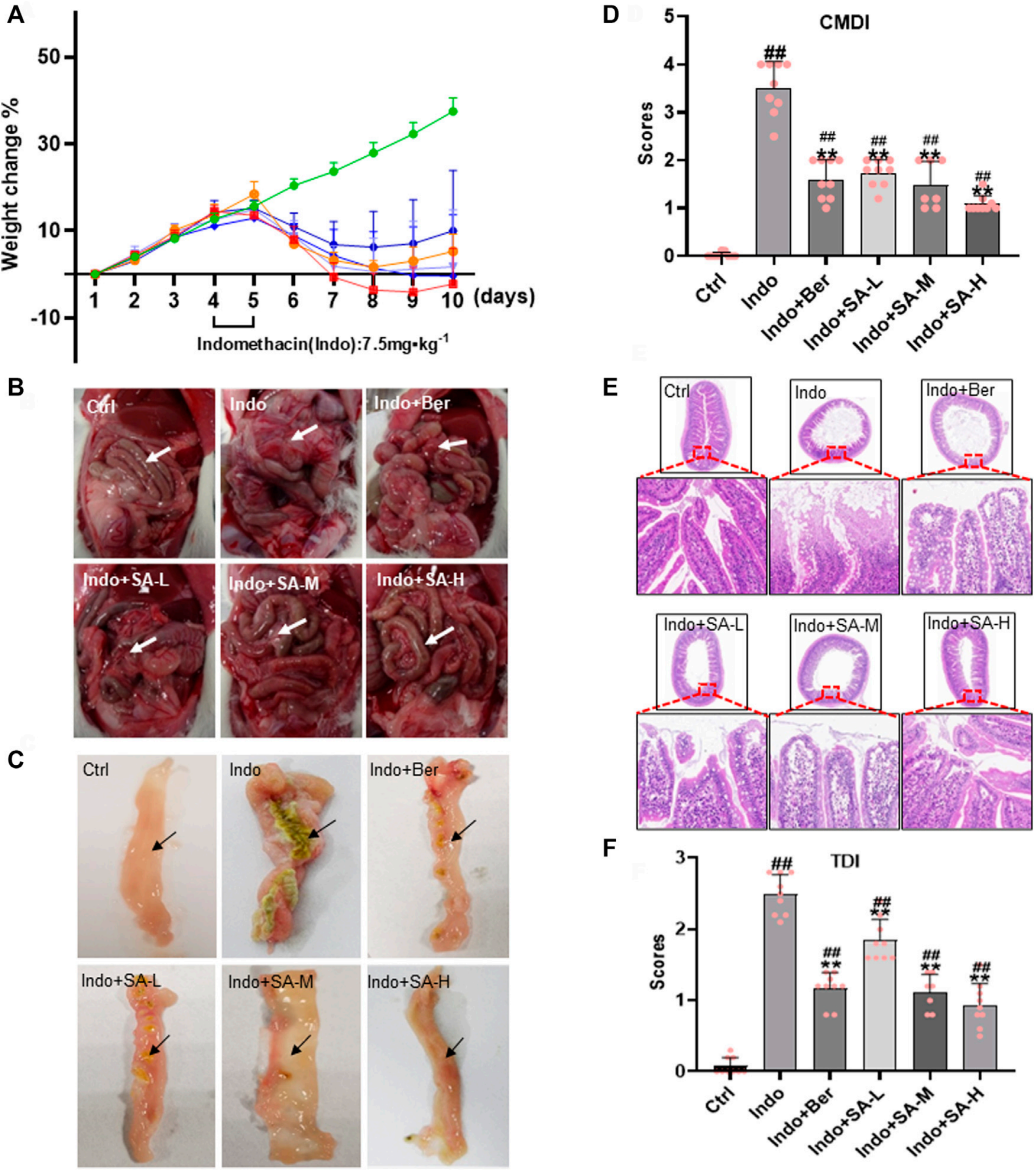
Protection of SA on small bowel inflammatory lesions induced by indomethacin in rats. Male SD rats at 180–200 g were treated with indomethacin (Indo, 7.5 mg kg^−1^) alone or with sanguinarine (SA, 0.33 mg kg^−1^, 1.0 mg kg^−1^, 3.3 mg kg^−1^, SA-L, SA-M and SA-H, respectively) or with berberine (Ber, 60 mg kg^−1^). Tissue sections of the jejunum and H&E (Hematoxylin and eosin) staining were prepared. **(A)** The body weight change of rats. **(B)** Gross damage of small intestine. **(C)** Jejunum intima macroscopy observations, showing mucosal lesions. **(D)** Colonic mucosal damage index (CMDI). **(E)** Representative images of jejunum histology (magnification at ×200). **(F)** Tissue damage index (TDI). Arrows indicate typical pathological changes. Data are presented as the mean ± SD (*n* = 9). ^#^ *p* < 0.05 and ^##^ *p* < 0.01 vs., control (Ctrl). *p < 0.05 and **p < 0.01 vs. indomethacin (Indo).

Weight change is an important indicator for intestinal inflammation and drug prevention and treatment of the inflammatory lesions in animal modeling (Yuan et al., 2019). In order to evaluate the protective effects of SA on indomethacin-induced small bowel inflammatory injury, SD rats with a body weight of 180–200 g were selected for modeling studies. The weight of rats was measured every day, and feeding and activity of rats were monitored daily. The results showed that control rats continued to gain weight with an average of 4% per day during the study period, but the indomethacin-treated rats stopped growth on the second day of drug delivery and then began to lose weight with 6% per day, reaching the peak on day 6. SA quickly reversed the effect of indomethacin on rat weight with a good dose-response relationship (Figure 1A). The effect of 1 mg kg^−1^ SA was similar to that of berberine at 60 mg kg^−1^.

At the same time, marked exudations, effusion, edema, erosion and even adhesion occurred in the abdominal cavity of rats in the indomethacin-treated group comparing to the control. However, these pathologic changes were significantly relieved in the rats that were administered with berberine or SA (Figure 1B). Similar changes were observed in the jejunum intima (Figure 1C) and CMDI scores (Figure 1D). The results showed that the jejunum intima in the control group was smooth without hyperplasia and adhesion, and that in Indo-treated group was swollen, enlarged with adhesion and serious peritoneal effusion. Berberine and SA could obviously protect the jejunum intima from Indo-induced injury in rats.

Small intestinal mucosal barrier (SIMB) plays an important role in NSAIDs-induced intestinal damage. Therefore, SIMB protection is the main strategy for alleviating intestinal injury in NSAIDS users (Lanza et al., 2009). In the indomethacin-treated rats, we observed obvious mucosal damage in the small intestine, including edema, inflammatory cell infiltration, shortened microvilli, abscission, and ulcers (Figures 1E,F). The microscopic pathological score, tissue damage index (TDI), was significantly increased. Interestingly, SA dose-dependently reduced the mucosal damage (Figure 1E) and improved the TDI (Figure 1F). No significant pathological changes of SIMB were observed in the control rats.

### Rescue by Sanguinarine of intestinal lactate dehydrogenase production and mucosal barrier in indomethacin-treated rats

**FIGURE 2:**
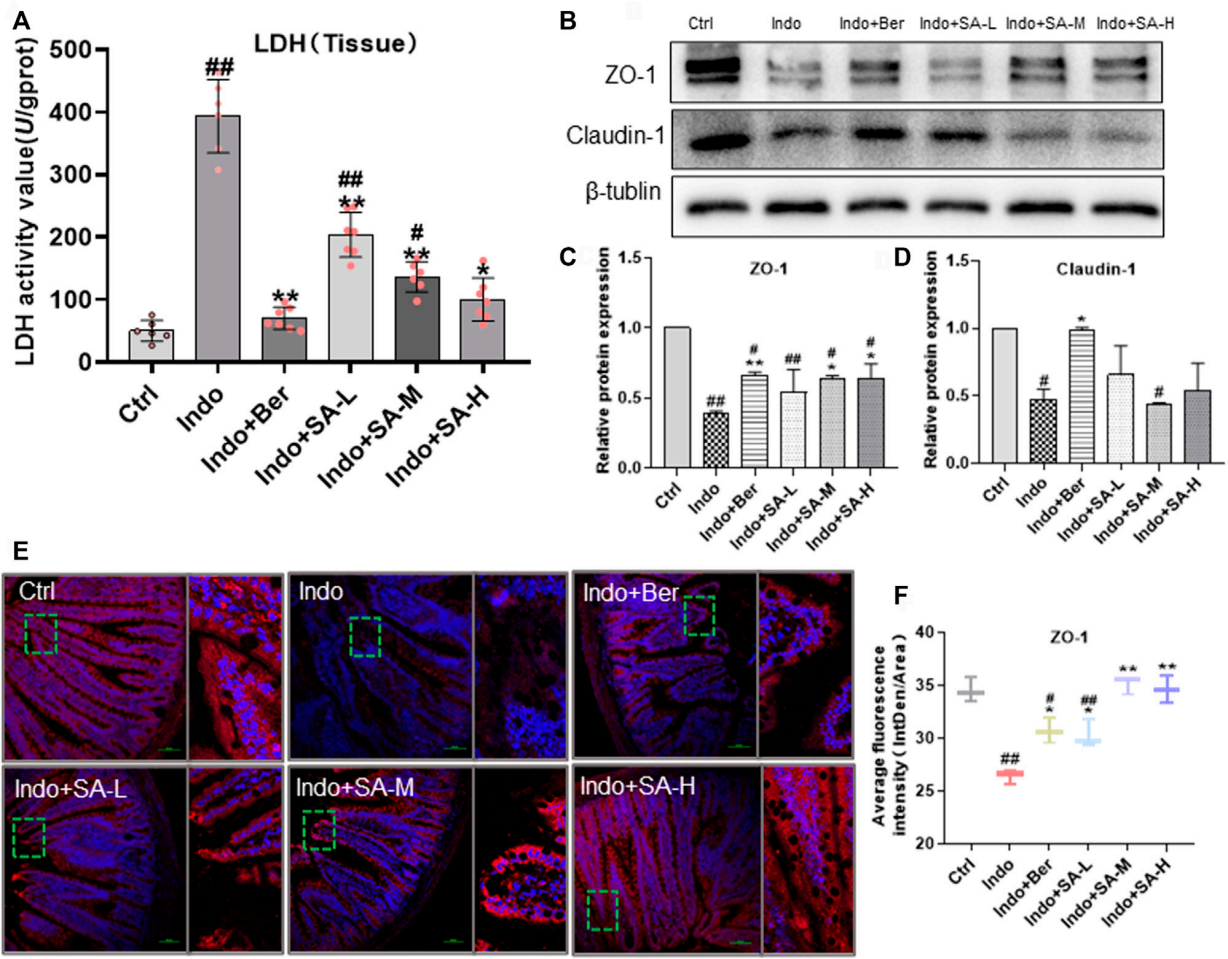
Protection of SA on mucosal barrier of jejunum in indomethacin-treated rats. Male SD rats were treated with indomethacin (Indo, 7.5 mg kg^−1^) alone or with sanguinarine (SA, 0.33 mg kg^−1^, 1.0 mg kg^−1^, 3.3 mg kg^−1^, SA-L, SA-M and SA-H, respectively) or with berberine (Ber, 60 mg kg^−1^).The jejunum tissue samples of SD rats were collected and prepared according to the requirements of the detection kit. Chemiluminescence was utilized to measure the levels of tissue lactate dehydrogenase (LDH) **(A)**. Western blot was used to measure the expression of tight junction proteins zonula occludens-1 (ZO-1) and claudin-1 **(B–D)**. The laser confocal immunofluorescence assay was used to detect the location and expression levels of ZO-1, and the small box represents the typical fluorescence area, which is enlarged and placed to the right of the whole fluorescence field **(E,F)**. Data are presented as the mean ± SD (*n* = 3). ^#^ *p* < 0.05 and ^##^ *p* < 0.01 vs. control (Ctrl). *p < 0.05 and **p < 0.01 vs. indomethacin (Indo).

Increase of tissue lactate dehydrogenase (LDH) is a key marker of tissue damage and permeability change of cell membrane (Filez et al., 1987). Small intestinal mucosal barrier (SIMB) is mainly composed of IECs and Tight Junctions (TJs) consisting of TJ proteins, such as zonula occludens 1 (ZO-1), claudin-1, occludin and muco-2 (Pearce et al., 2018; Chen L. et al., 2020). Recently, it has been reported that ZO-1 has special significance in maintaining the mucosal barrier function and permeability of intestinal epithelial mucosa (Yu et al., 2010). The levels of LDH and TJ proteins of intestinal tissues can effectively reflect the mucosal barrier function. In this study, Western blotting and immunofluorescent staining were used to detect the expression of TJ protein ZO-1 and claudin-1. Tissue LDH was measured by Colorimetry. Our results showed that indomethacin enhanced the tissue LDH levels, but SA decreased the LDH levels in a dose dependent manner (Figure 2A). At the same time, in the indomethacin-treated rats, the expression of ZO-1 and claudin-1 significantly decreased, but SA and berberine effectively increased the expression of ZO-1 without remarkable effect on claudin-1 (Figure 2B) which confirmed the Western blot data (Figures 2C,D). Therefore, we mainly observed the effect of SA on ZO-1 in Immunofluorescence staining. The fluorescence staining showed that indomethacin treatment decreased the average fluorescence intensity of ZO-1, and both Ber and SA could antagonize the effect of indomethacin (Figure 2E).

### Inhibition of Sanguinarine on indomethacin-induced intestinal inflammatory response and oxidative stress in rats

**FIGURE 3:**
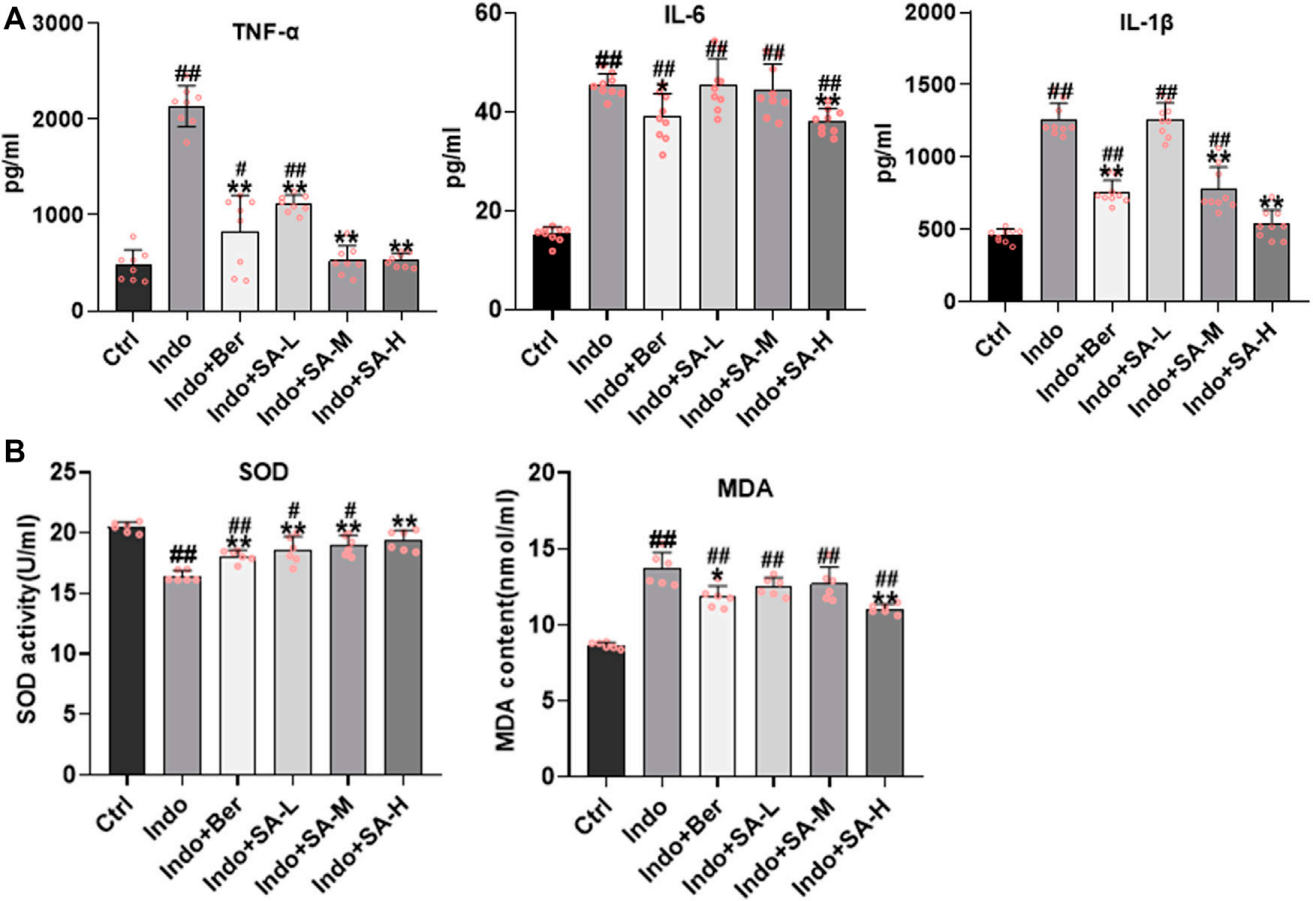
Inhibition of SA on indomethacin-induced intestinal inflammatory response and oxidative stress in rats. Male SD rats were treated with indomethacin (Indo, 7.5 mg kg^−1^) alone or with sanguinarine (SA, 0.33 mg kg^−1^, 1.0 mg kg^−1^, 3.3 mg kg^−1^, SA-L, SA-M and SA-H, respectively) or with berberine (Ber, 60 mg kg^−1^). The jejunum tissue of SD rats was collected and the supernatant was homogenized. After the protein was balanced, it was detected by ELISA, and the serum was detected by oxidative stress index. **(A)** Inflammatory cytokines. TNF-α (*left*), IL-6 (*middle*), and IL-1β (*right*) were measured by ELISA. **(B)** Oxidative stress. Super Oxide Dismutase (SOD) and Malondialdehyde (MDA) were estimated using a colorimetric assay kit and a chemiluminescent assay kit, respectively. Data are presented as the mean ± SD (*n* = 6). ^#^ *p* < 0.05 and ^##^ *p* < 0.01 vs. control (Ctrl). *p < 0.05 and **p < 0.01 vs. indomethacin (Indo).

It is well known that inflammatory response and oxidative stress are the key etiological elements in NSAIDs-induced intestinal damage. Many inflammatory factors, such as interleukins (IL), tumor necrosis factor α (TNF-α), chemokines (CKs), matrix metalloproteinases (MMPs), cyclooxygenases (COXs) and Interferon-γ (IFN-γ) are involved. In order to confirm that SA protected intestine mucosa through inhibition of inflammation and oxidative stress, we assessed the inflammatory factors (TNF-α, IL-6, and IL-1β) and oxidative stress factors (MDA and SOD) in jejunum tissues. The results showed that indomethacin significantly increased the expression of TNF-α, IL-6 and IL-1β in small intestine, but SA inhibited the expression of TNF-α, IL-6 and IL-1β (Figure 3A). Furthermore, results showed that SOD activity decreased but MDA increased in the intestinal mucosa of indomethacin-treated rats, but both berberine and SA significantly antagonized the effects of indomethacin (Figure 3B). The effects of SA showed a good dose-effect relationship.

### Effects by Sanguinarine on the expression of NF-κB, Keap-1, Nrf2 and HO-1 in small intestinal mucosa of indomethacin-treated rats

**FIGURE 4:**
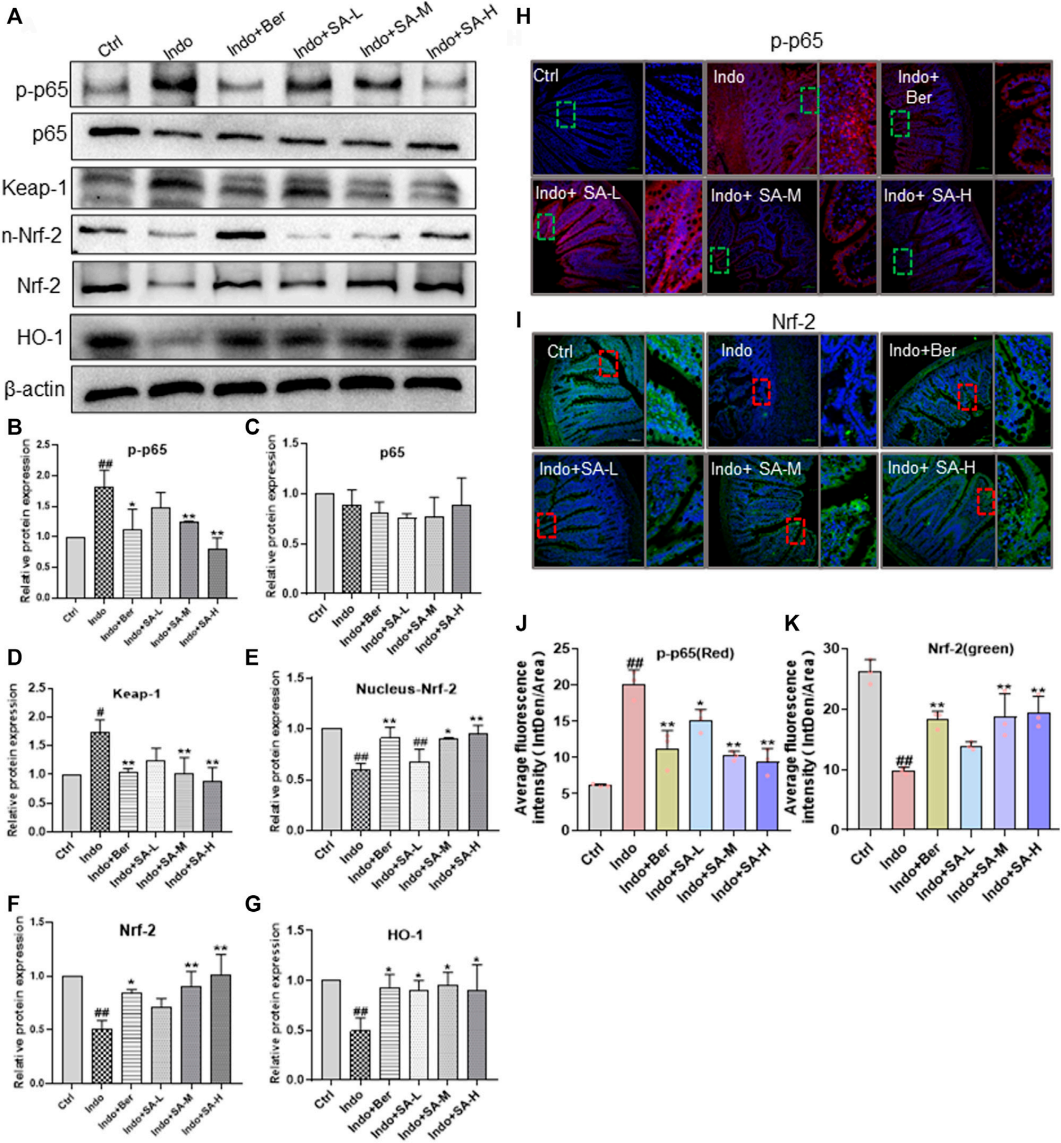
SA inhibited the NF-κB expression but increased Nrf2 and HO-1 expression in small intestinal mucosa of indomethacin-treated rats. Male SD rats were treated with indomethacin (Indo, 7.5 mg kg^−1^) alone or with sanguinarine (SA, 0.33 mg kg^−1^, 1.0 mg kg^−1^, 3.3 mg kg^−1^, SA-L, SA-M and SA-H, respectively) or with berberine (Ber, 60 mg kg^−1^). Protein was extracted from rat jejunum and tissue sections were prepared. **(A–G)** Protein levels of NF-KB p-p65, Keap-1, Nrf2, nucleus-Nrf2 and HO-1 evaluated by western blot with β-actin as an internal control. Data were calculated from at least three independent experiments. **(H-K)** Location and expressions of NF-kB p-p65 and Nrf2 in jejunum were detected by laser confocal immunofluorescence assay (magnification at ×200). NF-kB p-p65 and Nrf2 were labeled with FITC-red and FITC-green, respectively. The nuclei were stained with DAPI. The small box represents the typical fluorescence area, which is enlarged and placed to the right of the whole fluorescence field. Data are presented as the mean ± SD (*n* = 3). ^#^ *p* < 0.05 and ^##^ *p* < 0.01 vs. control (Ctrl). *p < 0.05 and **p < 0.01 vs. indomethacin (Indo).

NF-κB, Nrf2 and HO-1 play an important role in NSAIDs-induced small bowel injury (Wardyn et al., 2015). In order to elucidate the mechanisms by which SA antagonized indomethacin-induced intestinal mucosal inflammation, we analyzed the main inflammatory pathways and signals in NF-κB molecules involved. In this study, the protein levels of NF-κB p65 (p65), Keap-1, Nrf2, nucleus- Nrf2 and HO-1 were estimated by Western blot (Figures 4A–G) and laser confocal immunofluorescent analysis (Figures 4H–K). The results showed that indomethacin reduced the levels of Nrf2, nucleus- Nrf2 and HO-1 but enhanced the level of Keap-1 and phosphorylated NF-κB p-p65 (p-p65) (Figures 4A–G). In contrast, SA increased the expression of Nrf2 and HO-1, but decreased the expression of Keap-1 and p-p65 in a dose dependent manner (Figures 4A–G). SA had no marked effect on non-phosphorylated p65 (Figure 4C). The data from laser confocal immunofluorescence analysis showed that Indomethacin treatment enhanced the p-p65 level (Figure 4H, Figure 4J) with an increase of average fluorescence intensity and reduced the Nrf2 expression with a decrease of average fluorescence intensity. On the contrary, both Ber and SA could upregulate Nrf2 expression and decrease p-p65 level in a dose dependent manner (Figure 4I, Figure 4K).

### Effects of Sanguinarine on viability, permeability and barrier function of indomethacin-treated IEC-6 cells

**FIGURE 5:**
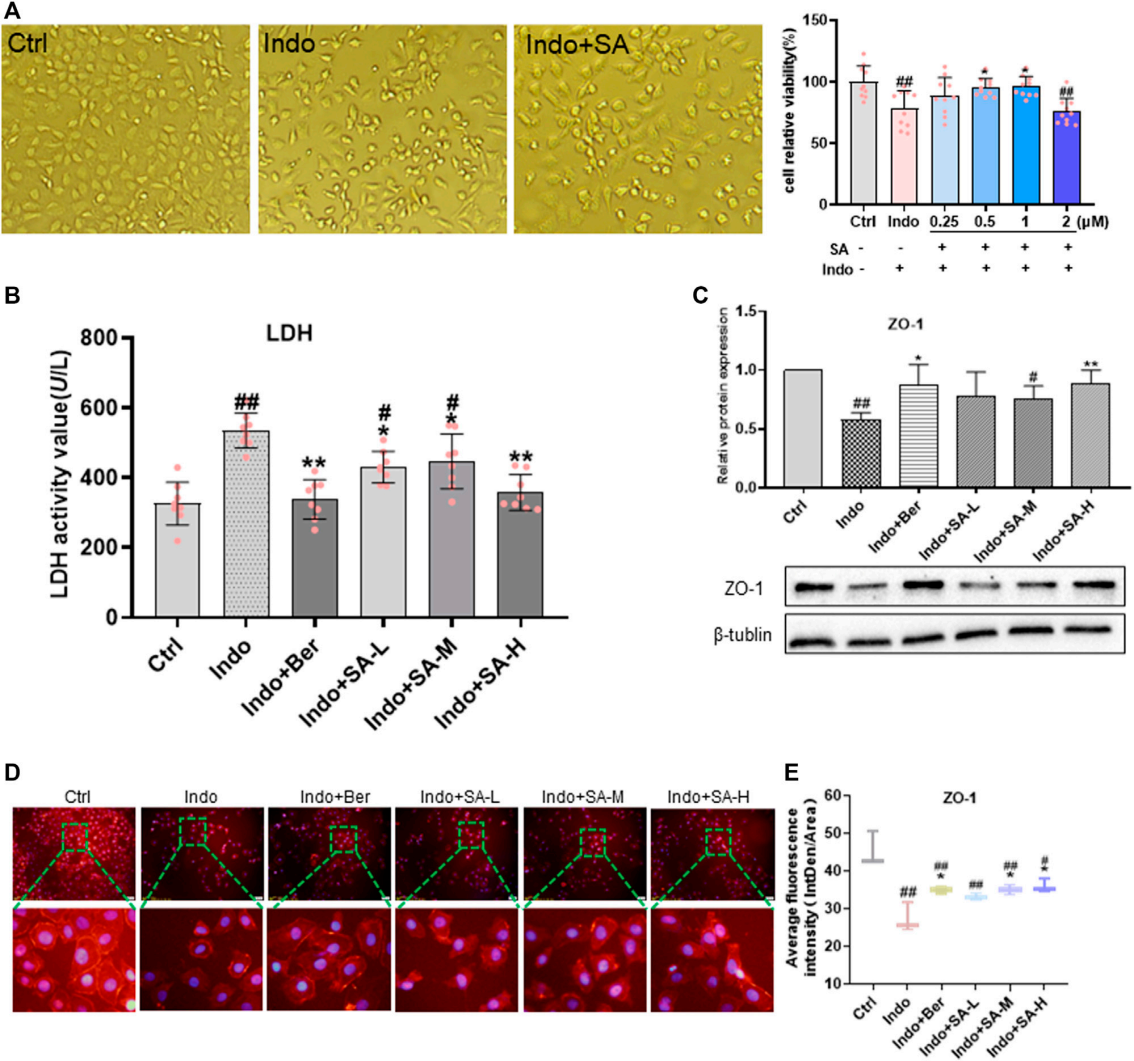
SA improved viability, permeability and barrier function of indomethacin-treated IEC-6 cells. IEC-6 cells were treated with indomethacin (Indo, 300 μmol L^−1^) and/or sanguinarine (SA, 0.25, 0.5, and 1.0 μmol L^−1^, SA-L, SA-M and SA-H, respectively) or with berberine (Ber, 30 mmol L^−1^), and cell viability was measured by CCK8 assay. **(A)** Viability of IEC-6 cells. Original pictures of IEC-6 cells (*left*, magnification at ×200); Protection of SA on indomethacin cytotoxicity (*right*). **(B)** LDH released from the IEC-6 cells, measured by chemiluminescence (*n* = 8). **(C)** Protein levels of ZO-1 estimated by western blot (*n* = 3). **(D,E)** Laser confocal immunofluorescent staining (*n* = 3). *Left*, images; *Right*, quantification. Data are presented as the mean ± SD. ^#^ *p* < 0.05 and ^##^ *p* < 0.01 vs. control (Ctrl). *p < 0.05 and **p < 0.01 vs. indomethacin (Indo).

Epithelial cell is a major component of the intestinal mucosal barrier (Vancamelbeke and Vermeire, 2017; Fan et al., 2021). To further evaluate the protection of SA on intestinal mucosa and to clarify the mechanisms of action, IEC-6 cells were used to estimate the effects of indomethacin and SA on epithelial cells (Figure 5A *left*). Furthermore, we observed that SA dose-dependently prevented IEC-6 cells damage induced by indomethacin at the range of 0.25–1.0 μmol L^−1^ (Figure 5A *right*). Therefore, indomethacin at 300 μmol L^−1^ and SA at 0.25–1 μmol L^−1^ were used in the subsequent experimental studies.

More importantly, in this study, we observed the effects of SA on membrane permeability and barrier function of IEC-6 cells demonstrated by LDH release and ZO-1 expression. As shown in Figure 5B, SA significantly inhibited the indomethacin-induced LDH release of IEC-6 cells but promoted ZO-1 expression (Figures 5C–E).

### Preventive role of Sanguinarine in inflammatory and oxidative stress in IEC-6 cells induced by indomethacin

**FIGURE 6:**
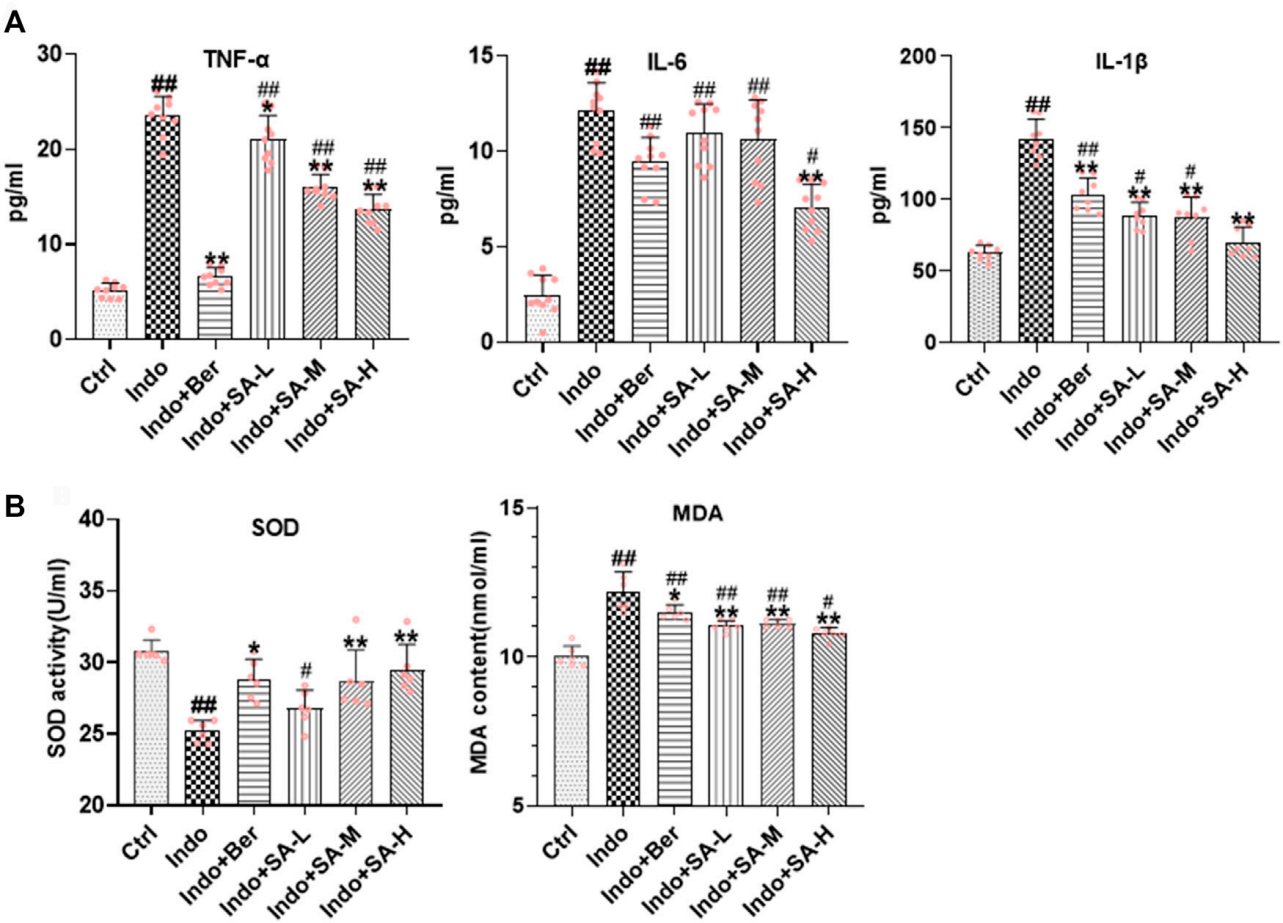
SA antagonized indomethacin-induced inflammatory and oxidative stress of IEC-6 cells. IEC-6 cells were treated with indomethacin (Indo, 300 μmol L^−1^) and/or sanguinarine (SA, 0.25, 0.5, and 1.0 μmol L^−1^, SA-L, SA-M and SA-H, respectively) or with berberine (Ber, 30 mmol L^−1^). **(A)** Inflammatory cytokines. TNF-α (*left*), IL-6 (*middle*), and IL-1β (*right*) were measured by ELISA (*n* = 8). **(B)** Oxidative stress. MDA and SOD were detected using a colorimetric assay kit and a chemiluminescent assay kit, respectively (*n* = 6). Data are presented as the mean ± SD. ^#^ *p* < 0.05 and ^##^ *p* < 0.01 vs. control (Ctrl). *p < 0.05 and **p < 0.01 vs indomethacin (Indo).

Both inflammatory and oxidative stress induced by NSAIDs can destroy barrier function and increase permeability of intestinal epithelial cells. To further confirm the protective role of SA in NSAIDs-induced damage of epithelial cells, we used IEC-6 cells to establish an indomethacin-induced inflammatory model, and found that SA remarkably inhibited the release of TNF-α, IL-6 and IL-1β (Figure 6A), reduced MDA levels, and promoted SOD activity (Figure 6B).

### Effects of Sanguinarine on expression of Keap-1, Nrf2, HO-1 and p-p65 induced by indomethacin in IEC-6 cells

**FIGURE 7:**
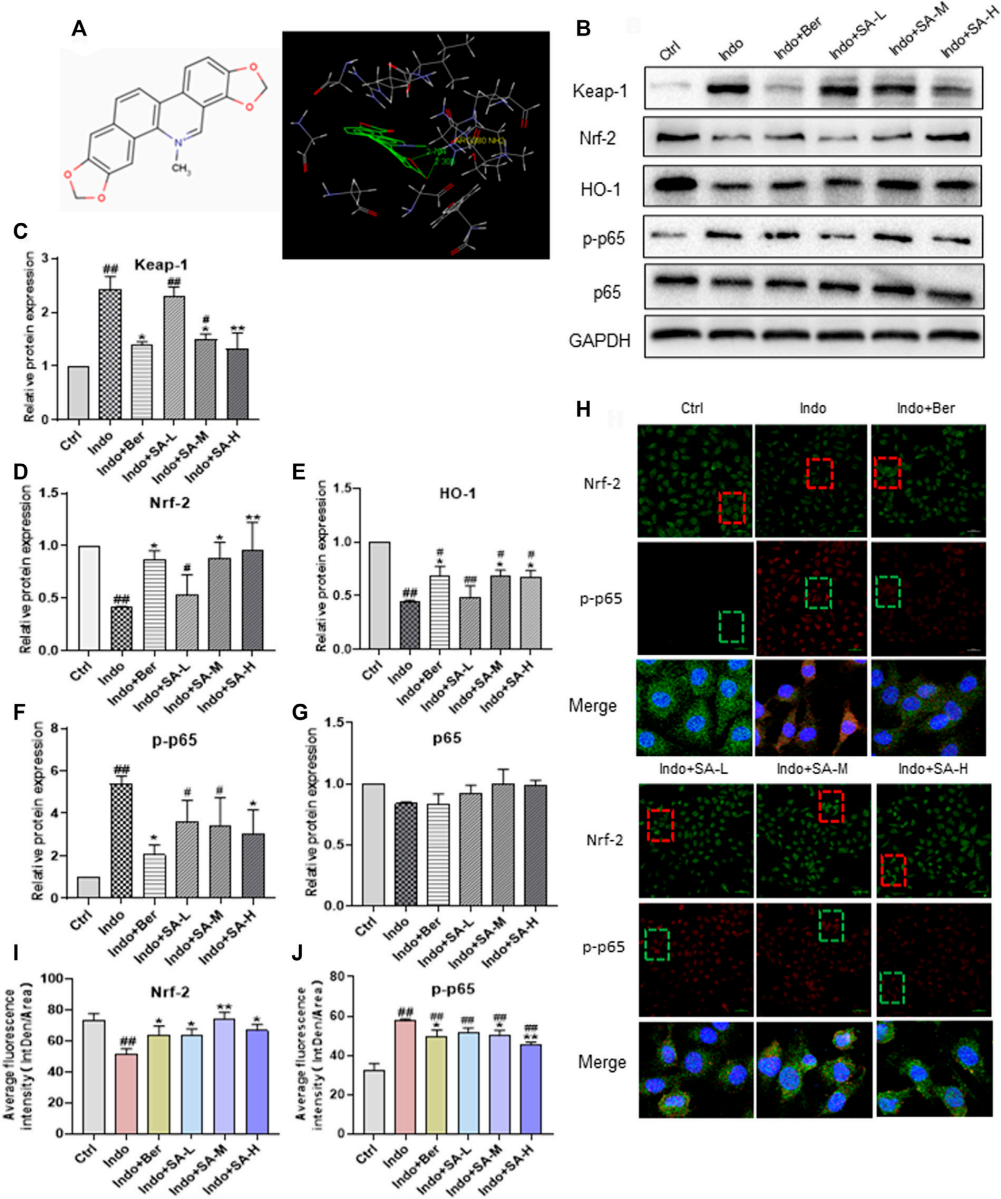
Binding of SA to Keap-1 to regulate the expression of Nrf2, p-p65, and HO-1 in indomethacin-treated IEC-6 cells. Molecular ligand docking was performed between SA and the KEAP1 using Gold 3.0. IEC-6 cells were treated with indomethacin (Indo, 300 μmol L^−1^) and/or sanguinarine (SA, 0.25, 0.5, and 1.0 μmol L^−1^, SA-L, SA-M and SA-H, respectively) or with berberine (Ber, 30 mmol L^−1^). Total cell protein and nuclear proteins of IEC-6 cells were extracted. **(A)** the structure of SA (left) and interaction of SA and Keap-1 (right). **(B–G)** Protein levels of Keap-1 increased, but decreased significantly in Ber and SA treatment groups. The expression of phosphorylated NF-kB p65 (p-p65), Nrf2 and HO-1 in IEC-6 cells, estimated by Western blot. **(H–J)** Expression of Nrf2 and p-p65 in IEC-6 cells shown by laser confocal immunofluorescence staining. Nrf2 and p-p65 were labeled with red and green FITC, respectively. The nuclei were stained with DAPI (magnification at ×200). Data are presented as the mean ± SD (*n* = 3). ^#^ *p* < 0.05 and ^##^ *p* < 0.01 vs. control (Ctrl). *p < 0.05 and **p < 0.01 vs. indomethacin (Indo).

It has been reported that Nrf2 is the target gene of Keap-1 (Baird and Yamamoto, 2020). In order to clarify the mechanism that sanguinarine (SA) regulates the Nrf2 pathway, we investigated the possible binding mode of SA to Keap-1. Molecular ligand docking *in silico* was performed between SA and the Keap-1 kelch domain using Gold 3.0. SA had a fully rigid structure, with six rings in the same plane, and the conformation occupied a small space (Figure 7A, left). Docking analysis showed that SA entered a large hydrophobic cavity and formed a hydrophobic interaction with Gly364, Tyr334 and Ala556. Two oxygen atoms in SA formed two hydrogen bonds with Arg380 (Figure 7A, right). These results suggest that SA could directly bind with Keap-1 protein and thereby has the potential for regulating Keap-1/Nrf2 pathway.

We further evaluated the expression of Keap-1**,** Nrf2, HO-1 and p-p65 (including total p65) in cultured IEC-6 cells in the presence of indomethacin with or without SA. As shown in Figure 7B, in the indomethacin-exposed cells, the expression of Keap-1 increased, but decreased significantly in Ber and SA treatment groups (Figure 7C). Meanwhile, indomethacin reduced the levels of Nrf2 and HO-1 proteins, accompanied by an increase of p-p65. SA promoted Nrf2 and HO-1 expression but downregulated p-p65 in a dose dependent manner (Figures 7D–G). Both indomethacin and SA had no effects on non-phosphorylated p65 (Figure 7E). Confocal immunofluorescence staining analysis further confirmed the effect of SA on the expression of Nrf2 and p-p65 in IEC-6 cells (Figures 7G,H).

### Effects of Sanguinarine on endonuclear expression and co-localization of Nrf-2, p-p65 and CBP in IEC-6 and the role of Nrf2 silencing

**FIGURE 8:**
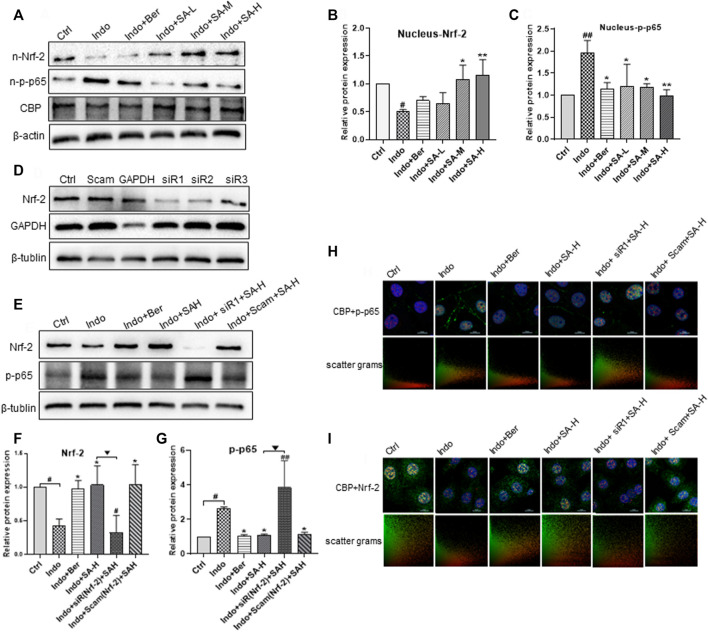
Regulation of SA on endonuclear expression and co-localization of Nrf2, p-p65 and CBP in IEC-6 and the effect of Nrf2 interference. IEC-6 cells were treated with indomethacin (Indo, 300 μmol L^−1^) and/or sanguinarine (SA, 0.25, 0.5, and 1.0 μmol L^−1^, SA-L, SA-M and SA-H, respectively) or with berberine (Ber, 30 mmol L^−1^). **(A–C)** Effects of SA on the expression of NRF-2, p-p65 and CBP in IEC-6. **(D)** Effects of different Nrf2 siRNAs on the Nrf2 levels of IEC-6 cells. IEC-6 cells were treated with 3 different Nrf2 siRNAs (50 nM), scrambled duplex (a blank control) or GAPDH siRNA (a positive control of RNA silencing). The expression of Nrf2 and GAPDH was measured by Western blot. **(E–G)** Effects of Nrf2 siRNA on the Nrf2 levels of IEC-6 cells at the present of indomethacin. **(H,I)** Effect of Nrf2 siRNA on location of Nrf2 and p-p65 in IEC-6 cells shown by laser confocal immunofluorescence staining. Nrf2 and p-p65 were labeled with green and red FITC, respectively. Nuclei were stained with DAPI. The upper right corner of the Merge diagram is the scatter plots of Nrf2 (green) and p-p65 (red), with the ordinate of Nrf2 and the abscis of p-p65. Data are presented as the mean ± SD (*n* = 3). ^#^ *p* < 0.05 and ^##^ *p* < 0.01 vs. control (Ctrl). *p < 0.05 and **p < 0.01 vs. indomethacin (Indo). ^▼^ *p* < 0.05 and ^▼▼^ *p* < 0.01 vs. Indo+SA-H.

It has been reported that the negative cross-talk between Nrf2 and NF-κB is related to competitive combination with transcriptional co-activator CBP complex (Liu et al., 2008). We proposed that anti-inflammatory effect of SA might be related to its regulation of competitive binding of Nrf2 or p-p65 with CBP. Therefore, we further detected endonuclear levels of Nrf2, p-p65 and CBP. The results showed that SA increased Nrf2 level in the nucleus and decreased the p-p65 level, but had no effect on CBP expression (Figures 8A–C). It is well known that Nrf2 and NF-κB counteract in regulation of redox state and inflammatory stress in cells (Cuadrado et al., 2014). In order to further confirm the action of SA on the expression of inflammatory factors through balancing the pathways of Nrf2 and NF-κB, we evaluated the effects of Nrf2 silencing on p-p65 expression and nuclear translocation using siRNA interference technology and confocal immunofluorescent staining assays. Our results showed that siRNAs targeting Nrf2 effectively decreased Nrf2 expression (Figure 8D). Meanwhile, the expression and nuclear entrapment of p-p65 increased significantly after Nrf2 silencing, and the inhibitory effect of SA and berberine on p-p65 expression was almost nullified by Nrf2 silencing (Figures 8E–G). More importantly, we observed the colocalization of Nrf2 with CPB and p-p65 with CBP by confocal fluorescence microscope. CBP was distributed in the nucleus in a dot shape. In ICE-6 cells exposed to indomethacin, the co-localization of Nrf2 and CBP decreased, while the co-localization of p-p65 and CBP increased. SA treatment could antagonize the effect of indomethacin, and the effect of SA was abolished by Nrf2 siRNA interference (Figures 8H,I).

### Effects of Nrf2 silencing on the expression of ZO-1, levels of inflammatory factors, super oxide dismutase activity and Malondialdehyde content of IEC-6 cells induced by indomethacin

**FIGURE 9:**
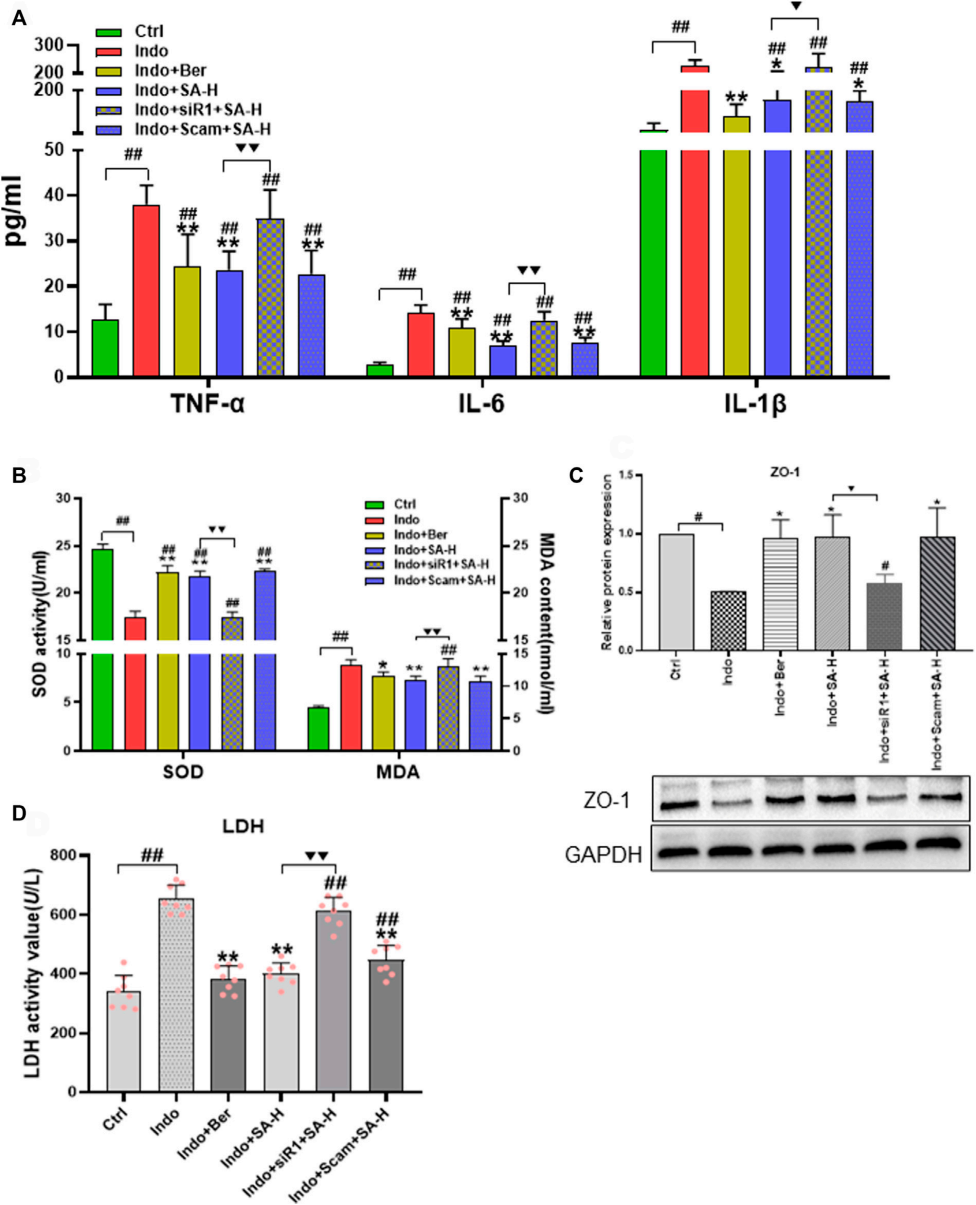
Effects of Nrf2 silencing on the expression of ZO-1, levels of LDH, inflammatory factors, SOD activity and MDA content of IEC-6 cells induced by indomethacin. IEC-6 cells were treated with indomethacin (Indo, 300 μmol L^−1^) and/or sanguinarine (SA, 1.0 μmol L^−1^, SA-H) or with berberine (Ber, 30 mmol L^−1^). **(A)** Inflammatory cytokines. TNF-α (*left*), IL-6 (*middle*), and IL-1β (*right*) were measured by ELISA (*n* = 9). **(B)** Oxidative stress parameters of MDA and SOD were detected using a colorimetric assay kit and a chemiluminescent assay kit, respectively (*n* = 6). **(C)** Protein levels of ZO-1 evaluated by western blot (*n* = 3). **(D)** LDH released from the IEC-6 cells, measured by chemiluminescence (*n* = 8). Data are presented as the mean ± SD. ^#^ *p* < 0.05 and ^##^ *p* < 0.01 vs. control (Ctrl). *p < 0.05 and **p < 0.01 vs. indomethacin (Indo). ^▼^ *p* < 0.05 and ^▼▼^ *p* < 0.01 vs. Indo+SA-H.

To further confirm the role of Nrf2 in SA protecting small intestine against NSAIDs injury, we investigated the effect of Nrf2 gene silencing on SA anti-inflammatory, anti-oxidation and protecting cellular barrier function in a model of IEC-6 cell injury induced by indomethacin. The results showed that SA-H (1.0 μ M) was found to significantly inhibit the release of inflammatory factors TNF-α, IL-6 and IL-1 β (Figure 9A). And the inhibitory effect of SA was blocked by Nrf2 silencing. SA decreased the level of MDA and promoted the activity of SOD, which were both significantly reversed by Nrf2 silencing (Figure 9B). Similarly, we observed the effect of SA on the membrane barrier function of IEC-6 cells, and found SA decreased LDH release and increased the level of membrane protein ZO-1. However, as shown in Figures 9C,D, the effects of SA on ZO-1 expression and LDH release were obviously reduced after Nrf2 silencing.

## Discussion

NSAIDs are extensively used in clinical practice, such as treating rheumatoid arthritis, for their prominent characteristics of anti-pyretic, anti-inflammatory and analgesic properties. However, some patients may experience serious gastrointestinal adverse effects, including dyspepsia, heartburn, peptic ulcer, bleeding, and perforation, etc. Patients often have to stop taking NSAIDs due to the side effects. For a long term, gastrointestinal damage induced by NSAIDs was thought to occur mainly in the colon, but in recent years, an increasing number of reports showed that NSAIDs could result in small intestine injuries (Graham et al., 2005; Maiden et al., 2005; Matsumoto et al., 2008). With the recent discovery that the small intestine is involved in the regulation of enterohepatic circulation (Chen et al., 2021), gut-brain Axis (Bauer et al., 2016), and especially, the host immune function (Kim et al., 2016; Schluter et al., 2020; Chen et al., 2021; Wastyk et al., 2021), the small intestine has also been emphasized as an therapeutic target for NSAIDs-induced gastrointestinal inflammation (Leung et al., 2007). The main pathological changes induced by NSAIDs are the damage of endothelial physical barrier due to NSAIDs-caused disorders of prostaglandin E synthesis, oxidative stress, inflammatory stress, immune dysregulation and intestinal microorganism dysbiosis.

In recent years, many drugs and health products are used to prevent and treat NSAIDs-induced gastrointestinal lesions. Misoprostol, a synthetic prostaglandin E1 analogue, was used as a first choice for treating low dose aspirin-induced enteropathy and showed to be effective on healing small-bowel ulcers in a randomized trial (Taha et al., 2018). Rebamipide, a muco-protective drug, could relieve NSAIDs-induced gastroduodenal damage (Kurokawa et al., 2014). Rifaximin, a poorly absorbed antibiotic, has a prophylactic effect against intestinal lesions induced by NSAID diclofenac (Fornai et al., 2016). Probiotics is another promising therapy to treat NSAIDs-induced enteropathy. Capsule endoscopic studies showed that both *Lactobacillus* casei (*L. casei)* and *Lactobacillus* gasseri (*L. gasseri)* could mitigate aspirin-induced small bowel injuries (Endo et al., 2011). In addition, colchicine (Otani et al., 2016), omeprazole (Maiden et al., 2005), infliximab and etanercept (TNF-α antagonist) (Watanabe et al., 2014), are also reported to relieve NSAIDs-induced small intestinal damage. However, the clear effects and mechanisms of these drugs remain unclear (Watanabe et al., 2020).

Botanical drugs have obvious advantages in alleviating the small intestinal injuries caused by NSAIDs. It has been reported that berberine, curcumin, and quercetin could relieve gastrointestinal injuries of inflammatory bowel disease (IBD) patients or NSAIDs users (Fan et al., 2021; Wang et al., 2021). SA is one of the main alkaloids (isoquinoline alkaloid) in the *Macleaya cordata* extracts (MCE) (Qing et al., 2021). In our previous studies, we systematically investigated the source (Huang et al., 2018; Qing et al., 2021) and detection methods Chen et al., 2009) of SA, analyzed biosynthetic pathway of SA in Macleaya cordata (Liu et al., 2017) and its pharmacokinetics following oral and intravenous administration (Wu et al., 2013; Hu et al., 2019). It has been reported that SA has a strong pharmacological activity, such as enhancing innate immunity (Guan et al., 2019), protecting intestinal mucosa and antioxidation (Liu et al., 2016; Guan et al., 2019). Four principal isoquinoline alkaloids, such as SA, chelerythrine, allocryptopine, chelionine, were found in the MCE (Huang et al., 2018). Among them, SA possesses strong anti-tumor, anti-inflammatory and antioxidant activities, as well as promoting animal growth (Ahmad et al., 2000; Rahman et al., 2016; Palócz et al., 2019; Liu et al., 2020). SA is a potent inhibitor of NF-κB (Chaturvedi et al., 1997), mitogen-activated protein kinase (MAPK) phosphatase-1 (Vogt et al., 2005) and Akt signaling cascade (Rahman et al., 2016), as well as a strong protective agent of HO-1 (Park et al., 2014; Vrba et al., 2012). It has been reported that SA could inhibit acetic acid-induced ulcerative colitis by regulating the MAPK/NF-κB pathway in mice (Niu et al., 2013), mitigate inflammatory responses in porcine jejunal cells (Palócz et al., 2019), and enhance growth performance in broilers by modulating gut microbiome (Liu et al., 2020). However, whether SA can relieve small intestinal inflammatory injury induced by NSAID has not been reported.

In the present study, we discovered that SA recovered the body weight lost by indomethacin in rats and drove a series macroscopic relieves of small intestine, e.g., intimal ecchymosis, bleeding, edema, exudation, congestion, adhesion, and CMDI scores (Figure 1). Histologically, SA significantly reduced the infiltration of inflammatory cells and ulcer formation of small intestinal mucosa as shown by H&E staining (Figure 1). Meanwhile, SA protected intestinal barrier function by promoting ZO-1 expression, increasing SOD activity, declining of LDH release and MDA oxidative marker, and inhibiting the expression of TNF-a, IL-1β and IL-6 in indomethacin-treated rats and IEC-6 cells (Figures 2, 3, 5, 6). Notably, SA inhibited p65 phosphorylation and enhanced the levels of Nrf2 and HO-1 in both indomethacin-treated rats and IEC-6 cells (Figures 4, 7).

The integrity of the structure and function of the small intestinal intima (especially the barrier function) is the basis of intestinal resistance to inflammation and oxidative stress. The small intestinal intima barrier mainly includes biological, immune, chemical and physical barriers. It is well known that tight junctions and membrane permeability between intestinal endothelial cells are the main components of the biological barrier, which play an important role in preventing intestinal inflammatory injury. Recently, Chen et al. (2021) has reported that the small intestine is a specific place where the immune cells, such as CD4 +T cell, prevent against inflammation. They found that CD4 +T effector (Teff) cell need to infiltrate the small intestine lamina propria (siLP), not the colon, for activating CAR/MDR1 pathway, initiating transcriptional reprogram and sub-specialization. Here the Teff cells detoxify bile acids and resolve inflammation. Based on the critical role of small intestinal intima in maintaining the integrity and stability of small intestine lamina propria, our study focused on the antagonistic effect of SA on the biological barrier injury of small intestinal intima and endothelial cell (IEC-6) caused by indomethacin. Our results showed that SA could effectively protect the permeability stability of small intestinal intima and endothelial cell membrane, increase the expression of TJ protein ZO-1, and reduce the release of LDH induced by indomethacin (Figures 2, 5B–E).

NF-κB is a major nuclear transcription factor which regulates the transcription of inflammatory factors, such as IL-1β, IL-6, TNF-α and COX-2 (Vallabhapurapu and Karin, 2009) and is considered as an ideal target for the treatment of NSAIDs-induced gastrointestinal injuries. It has been shown that in patients with IBD or NSAID-induced gastrointestinal damage, the NF-κB signaling pathway was always abnormally activated. NF-κB is a heterodimer composed of p50 and p65. Normally, NF-κB is located in the cytoplasm in a complex with IκB (an inhibitory protein of NF-κB) (Scheidereit, 2006). When the body or cell is exposed to exogenous substances (e.g., LPS or indomethacin) or proinflammatory cytokines (e.g., TNF-α and IL-1β), IκB is phosphorylated by IκB kinase (IκK) and release NF-κB. The activated NF-κB is then translocated into the nucleus, binds with specific genes and promotes transcription of TNF-α, IL-1β, IL-6, etc. Our results showed that SA significantly reduced the level of p-p65 in both cytoplasm and nucleus, but not on non-phosphorylated p65 (Figure 8).

It is well known that Nrf2 mainly regulates the expression of antioxidant proteins, such as HO-1 (DeNicola et al., 2011). In recent years it is found that Nrf2 also plays a key role in inflammatory disease including IBD (Ahmed et al., 2017; Mills et al., 2018). Under physiological conditions, Nrf2 is primarily located in the cytoplasm combining with Keap-1 (a inhibiting protein of Nrf2) and maintains at a low level through Keap-1 E3-mediated degradation (Baird and Yamamoto, 2020). Under the stimulation of ROS, and/or inflammatory cytokines, Keap-1 mediated Nrf2 degradation is terminated and cytoplasm Nrf2 is accumulated. Nrf2 is then translocated into the nucleus, binds to antioxidant response element ARE, and regulates expression of antioxidant proteins, such as HO-1, participating in Nrf2-mediated NF-κB inhibition. In the present study, we found that indomethacin decreased the expression of Nrf2 and HO-1 in both rats and IEC-6 cells, while SA markedly increased the level of Nrf2 and HO-1 in a dose dependent manner (Figures 4, 7).

Nrf2 and NF-κB are key regulators for the balance of cellular redox status and for r inflammatory response through a negative crosstalk. Many studies showed the disorders of Nrf2 and NF-κB signaling pathways in IBD patients or NSAIDs-induced gastrointestinal damage, and Nrf2 activation was involved in the protective effect on indomethacin-induced damage of IEC-6 cells (Harada et al., 2015; Wardyn et al., 2015; Sivandzade et al., 2019). Therefore, we proposed that the anti-inflammatory effects of SA on NSAIDs-induced enteritis could be related to the regulation of Nrf2 and NF-κB interplay. In order to confirm our hypothesis, we evaluated the levels of Nrf2 and p-p65 in SA-treated IEC-6 cells with siRNA interference using immunofluorescence co-localization. We found that SA induced Nrf2 expression and decreased the level of PP65, and the effect of SA is cancelled by Nrf2 silencing (Figure 8). In addition,Nrf2 silencing almost completely counteracted the protective effect of SA on barrier function, as well as anti-inflammatory and anti-oxidation effect of SA in indomethacin-treated IEC-6 cell (Figure 9). More importantly, the effect of SA on regulating colocalization of Nrf2 with CPB and p-p65 with CBP was abolished by Nrf2 siRNA interference (Figures 8H,I). These findings further confirmed that Nrf2 mediated the protective effect of SA against intestinal inflammatory injury.

**FIGURE 10:**
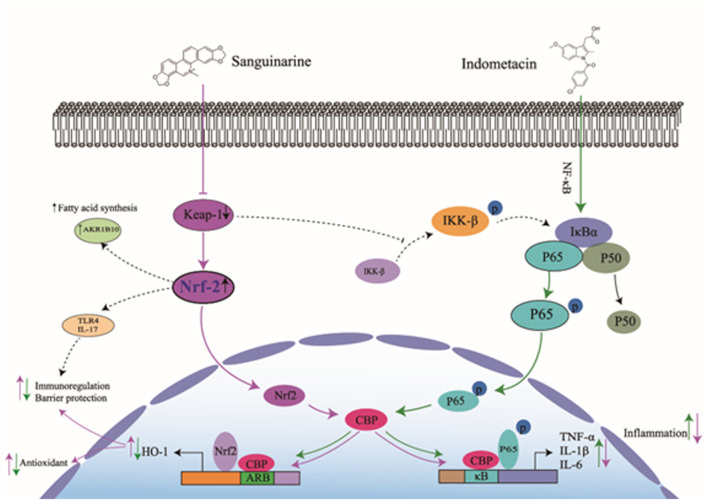
Hypothetic model of SA protection from NSAIDs-indued inflammatory damage of small intestine through regulating the Nrf2/NF-KB pathways. Under the stimulation of NSAIDs and other inflammatory factors, the inflammatory pathway of NF-kB is activated in epithelial cells and intestinal tract. P65 (an active subunit of NF-KB) is phosphorylated and depolymerized from the inflammatory complex composed of p65, p50 and IκB-α. Then phosphorylated p65 (p-p65) enters the nucleus, binds with its target gene under the guidance of CBP, and induces the expression of inflammatory factors TNF-α, IL-6 and IL-1β, which activate the inflammatory response of IEC-6 cells and intestinal mucosa, leading to inflammatory lesions. SA binds to Keap-1, inhibits Keap-1 and increases cellular expression of Nrf2, which competes with p-p65 to bind to CBP in the nucleus, and then reduce the expression of TNF-α, IL-6 and IL-1β. On the other hand, the increased Nrf2/CBP complexes promote the expression of HO-1 (target gene of Nrf2), so as to counteract the effects of inflammatory factors TNF-α, IL-6 and IL-1β.

Nrf2 regulates NF-κB activity by multiple pathways: such as modulating p65 activity by interplay with MafK (a s Maf proteins) (Hwang et al., 2013), increasing intracellular GSH levels (Ganesh Yerra et al., 2013), increasing HO-1 expression and competitively combining with CBP. The most well-established negative cross-talk between Nrf2 and NF-κB is a competitive combination with CBP–p300 complex (Liu et al., 2008). CBP is a transcriptional co-activator that helps transcription factors, such as p65 and Nrf2, to initiate transcription of target genes, such as HO-1, TNF-α, and IL-1. CBP has intrinsic histone acetyltransferase activity which causes histone acetylation and loosens the chromatin structure. Thus, DNA is exposed to transcription factors, such as p-p65 and Nrf2 (Ganesh Yerra et al., 2013). When Nrf2 combines with CBP, it forms a complex with ARE to promote the transcription of Nrf2 targeted gene HO-1. On the other hand, when CBP binds to p-p65 (at Ser276), it forms a complex with MafK and promotes the transcription of p-p65 targeted genes, such as TNF-α, IL-6, and IL-1β (Vanden Berghe et al., 1999; Hwang et al., 2013). In this study, we confirmed that upregulated Nrf2 may increase its binding to CBP, thus depriving CBP interaction with p65 and thereby decreasing expression of TNF-α, IL-6 and IL-1β (Figure 10).

## Conclusion

We demonstrated that SA significantly prevented indomethacin-induced intestinal damage in rats and IEC-6 cells by inhibiting inflammatory and oxidative stress, protecting intestinal barrier function. Importantly, we discovered for the first time that the entero-protection of SA was mediated through balancing the Nrf2 and NF-κB pathways. Our study provides a new mechanism accounting for the protective role of SA in NSAID-induced intestinal damage.

## Data Availability

The original contributions presented in the study are included in the article/supplementary material, further inquiries can be directed to the corresponding authors.

## Abbreviations

SA, sanguinarine; Indo, indomethacin; Ber, berberin; CMDI, colonic mucosa damage index; TDI, tissue damage index; SIMB, small intestinal mucosal barrier; TJs, tight junctions; ZO-1, zonula occludens 1.
